# Regulation of Geldanamycin Biosynthesis by Cluster-Situated Transcription Factors and the Master Regulator PhoP

**DOI:** 10.3390/antibiotics8030087

**Published:** 2019-06-30

**Authors:** Juan F. Martín, Angelina Ramos, Paloma Liras

**Affiliations:** 1 Area de Microbiología, Departmento de Biología Molecular, Universidad de León, 24071 León, Spain; 2 Instituto de Biotecnología (INBIOTEC). Av. Real 1, 24006 León, Spain

**Keywords:** geldanamycins, *Streptomyces hygroscopicus*, antitumorals, biosynthesis, gene cluster, phosphate control

## Abstract

Geldanamycin and the closely related herbimycins A, B, and C are benzoquinone-type ansamycins with antitumoral activity. They are produced by *Streptomyces hygroscopicus* var. *geldanus*, *Streptomyces lydicus* and *Streptomyces autolyticus* among other *Streptomyces* strains. Geldanamycins interact with the Hsp-90 chaperone, a protein that has a key role in tumorigenesis of human cells. Geldanamycin is a polyketide antibiotic and the polyketide synthase contain seven modules organized in three geldanamycin synthases genes named *gdmAI*, *gdmAII*, and *gdmAIII*. The loading domain of GdmI activates AHBA, and also related hydroxybenzoic acid derivatives, forming geldanamycin analogues. Three regulatory genes, *gdmRI*, *gdmRII*, and *gdmRIII* were found associated with the geldanamycin gene cluster in *S. hygroscopicus* strains. GdmRI and GdmRII are LAL-type (large ATP binding regulators of the LuxR family) transcriptional regulators, while GdmRIII belongs to the TetR-family. All three are positive regulators of geldanamycin biosynthesis and are strictly required for expression of the geldanamycin polyketide synthases. In *S. autolyticus* the *gdmRIII* regulates geldanamycin biosynthesis and also expression of genes in the elaiophylin gene cluster, an unrelated macrodiolide antibiotic. The biosynthesis of geldanamycin is very sensitive to the inorganic phosphate concentration in the medium. This regulation is exerted through the two components system PhoR-PhoP. The *phoRP* genes of *S. hygroscopicus* are linked to *phoU* encoding a transcriptional modulator. The *phoP* gene was deleted in *S. hygroscopicus* var *geldanus* and the mutant was unable to grow in SPG medium unless supplemented with 5 mM phosphate. Also, the *S. hygroscopicus* *pstS* gene involved in the high affinity phosphate transport was cloned, and PhoP binding sequences (PHO boxes), were found upstream of *phoU*, *phoRP*, and *pstS*; *the phoRP-phoU* sequences were confirmed by EMSA and nuclease footprinting protection assays. The PhoP binding sequence consists of 11 nucleotide direct repeat units that are similar to those found in *S. coelicolor Streptomyces avermitilis* and other *Streptomyces* species. The available genetic information provides interesting tools for modification of the biosynthetic and regulatory mechanisms in order to increase geldanamycin production and to obtain new geldanamycin analogues with better antitumor properties.

## 1. Introduction: Antitumor Activity of Geldanamycin and its Derivatives

**Figure 1:**
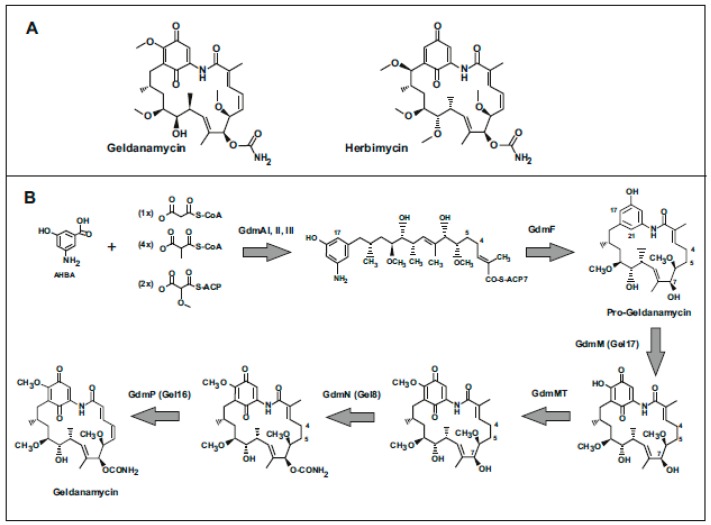
Geldanamycin structure and biosynthesis pathway. (**A**) Structures of geldanamycin and herbimycin. (**B**) Geldanamycin biosynthetic pathway. The enzymes involved in every step are indicated.

Geldanamycin and the closely related herbimycins A, B, and C are benzoquinone-type ansamycins [1] with antitumoral activity [2,3] (Figure 1A). These compounds are produced by different strains of *S. hygroscopicus*, *Streptomyces lydicus*, and by *Streptomyces autolyticus*, and gene clusters for geldanamycin have been found in other *Streptomyces* strains (Table 1). Both, geldanamycin and the herbimycins were discovered as weak antifungal and antibacterial antibiotics [4,5,6] but their major interest is as potent antitumor agents due to their ability to interact with the Hsp-90 chaperone complex in human cells [7,8]. The members of the Hsp-90 chaperone family play an important role in the tumorigenesis process in humans. Both geldanamycin and the herbimycins have potent antitumor activity at nanomolar concentration, particularly the former [9,10]. However, it was shown that the natural compounds are hepatotoxic [11]. In the last decades, great interest has focused on the discovery of new derivatives with lower toxicity by direct genetic modification of the known geldanamycin gene cluster and investigation on new producer strains [3,12,13,14]. Structurally similar compounds, such as 17-amino-17-demethoxy-geldanamycin, were found in a knock out mutant of *S. autolyticus* CGMCC 0516 [15]. Two other chemical derivatives of geldanamycin, 17-allylamino-17-demethoxygeldanamycin, and 17-(2-dimethylamino) ethylamino-17-demethoxy-geldanamycin have been tested in clinical trials [16,17].

## 2. Biosynthesis of Geldanamycins

Geldanamycin is a polyketide derived ansamycin [4] that is synthesized from precursors assembled by polyketide synthases. In general, the geldanamycin biosynthetic process can be divided in three large steps: (1) Biosynthesis of the precursor 3-amino-5-hydroxybenzoic acid (AHBA); (2) extension of the starter unit with polyketide elongation units, and (3) post-polyketide modifications [19]. 

### 2.1. Origin and Biosynthesis of the AHBA Unit 

All ansamycins derive from a seven carbon and one nitrogen atom (mC_7_N) AHBA starter unit, that is elongated using malonyl-CoA or methylmalonyl-CoA units and finally form the ansamycin ring through a lactam bond. The AHBA unit is synthesized from glucose by the amino shikimate pathway which includes the three initial steps similar to those of the shikimate pathway (for the biosynthesis of aromatic amino acids). Previous information on the synthesis of the AHBA unit of rifamycin provides evidence indicating that seven genes, *rifG*HJKLMN, linked in a cluster, are required and sufficient to synthesize this compound in *Amycolatopsis mediterranei*. The gene *rifK* of this cluster, that encodes the AHBA synthase, has been extensively used to search for ansamycin gene clusters in other actinobacteria [25,26,27,28]. Homologs of these genes were searched in *S. hygroscopicus* 17997, producer of geldanamycin, and two different gene clusters were found. One of them, of the benzoquinone type, was shown to be involved in geldanamycin biosynthesis [18]. Genes of the second cluster, of the naphtoquinone type, did not complement mutants in geldanamycin biosynthesis in *S. hygroscopicus* [19]. 

The work of He et al. [19] provides evidence showing that the benzoquinone-type cluster in the *S. hygroscopicus* strain is, indeed, involved in geldanamycin biosynthesis while the naphtoquinone-type cluster is likely to be involved in biosynthesis of a rifamycin-type ansamycin. Similarly, two gene clusters encoding benzoquinone-type and naphtoquinone-type ansamycins have been found in *Streptomyces collinus* that produces ansatrienin and naphthomycin [26]. The presence of two related gene clusters in single *Streptomyces* species is relatively common. This is normally generated by gene duplication and subsequent specialization. Interestingly, there are differences in the organization of the AHBA gene cluster in the original *S. hygroscopicus* NRRL 3602 [18] and *S. hygroscopicus* 17997 [19]. This indicates that these two strains have evolved separately in modern times, although most likely both AHBA gene clusters derive from a common cluster in an ancestor of both strains. 

### 2.2. Elongation Steps

**Figure 2:**
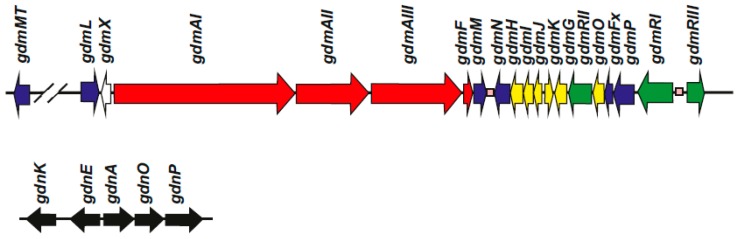
Cluster of geldanamycin genes in *S. hygroscopicus* NRRL3602. Cluster of geldanamycin genes in *S. hygroscopicus* NRRL3602 [18]. The name of each gene is indicated above it. Similar gene clusters have been found in other *S. hygroscopicus* strains and in *S. autolyticus* with the minor differences described in the text. Genes for the geldanamycin precursor AHBA [19], named *gdnKEAOP*, and located separately in the genome, are shown below. The names of the geldanamycin genes given by different authors and function of the encoded enzymes are shown in Table 2.

After formation of the AHBA starter unit the biosynthesis of geldanamycin proceeds by elongation steps involving one malonyl-CoA unit, 4 methylmalonyl-CoA units, and 2 methoxy-malonyl-ACP elongation units. These elongation reactions are catalyzed by three polyketide synthases encoded by *gdmAI*, *gdmAII*, and *gdmAIII*, totaling seven modules. They are organized in three polyketide synthases (Figure 2, Table 2), containing the AT, KS, ACP, and KR domains. The first condensation step catalyzed by a PK type I is of interest, since it includes a loading domain in addition to the ACP domain of the PKS. The loading domain of this PKS activates AHBA and related aromatic units [29] by a mechanism that uses ATP and is similar to that performed by the A domain of NRPSs [30,31]. In this reaction the carboxyl group of the AHBA is activated as an acyl-phosphate and then is transferred to the ACP1 in the first PKS synthase. This activation and elongation is similar to the p-aminobenzoic acid (PABA) activation and elongation performed by the first module of the candicidin PKS [32]. Noteworthy, the loading domain has low substrate specificity and is able to activate several benzoic acid related compounds. This activation and first elongation are also very similar to those performed by the rifamycin producer LM-M1 bimodule of *A. mediterranei*. In this strain the protein encoded by the first PKS, named *rifA*, served as a model for the biosynthesis of rifamycin and other ansamycins in different *Streptomyces*; it contains an initial didomain consisting of an AHBA activating A domain and an ACP domain. This didomain, separated from the rest of the PKS protein, has been expressed in *E. coli* and shown to be able to activate AHBA and related benzoic acid derivatives [29]. The requirement of ATP and CoA in the in vitro (*E. coli* extracts) reaction excludes the possibility that the starter unit is provided to the PKS in an activated different form during its synthesis in the shikimate pathway. This loading enzyme is able to activate, in addition to the natural starter unit, other 3- or 5-substituted benzoic acid derivatives and 3,5-disubstituted derivatives. Therefore, as occurs in the case of rifamycin, the loading module in the GdmA PKS of the geldanamycin gene cluster has the potential to synthesize compounds similar to geldanamycin containing aromatic units related to AHBA. The subsequent elongation steps use the standard polyketide biosynthesis mechanisms. 

Transcriptional studies of the geldanamycin polyketide synthase genes in *S. hygroscopicus* XM201 suggested that expression of the polyketide synthase genes was limiting for geldanamycin biosynthesis [21]. These authors replaced the native polyketide synthase promoter upstream of *gdmA1* by a strong endogenous *S. hygroscopicus* XM201 promoter selected on the basis of its high transcriptional activity. Interestingly, the replacement of the native promoter led to overexpression of the *gdmA* genes (4 to 141-fold) leading to 39% increase of geldanamycin production. Then, in this overproducing strain, biosynthesis of the AHBA starter unit became limiting. Combined overexpression of the polyketide synthase gene and the amino shikimate gene cluster resulted in an increase of 88% in geldanamycin production [21].

### 2.3. Cyclization of the Lineal Polyketide to Progeldanamycin 

After completion of the growing polyketide chain, it is released from the enzyme by intramolecular cyclization catalyzed by the amide synthase encoded by *gdmF*, a gene present in all geldanamycin producers (Figure 2, Table 2). It is noteworthy that the amide synthase of the geldanamycin gene cluster of *S. hygroscopicus* has much broader substrate specificity than the homologous enzyme of *Actinnosynnema pretiosum* [33], that produces ansamitocin, a cytotoxic compound. These authors isolated mutants of each of these two microorganisms blocked in the synthesis of the AHBA starting unit, to test whether they are able to incorporate other hydroxybenzoic acid compounds (which lacks the amino group of AHBA). Using these mutants, they demonstrated that at the difference of the *A. pretiosum* amide synthase, the enzyme of *S. hygroscopicus* was able to cyclize the linear polyketide intermediate containing hydroxybenzoic acid, forming a 20 membered lactone ring instead of the standard 19 membered lactam ring of geldanamycin. The difference of the amide synthases in these two organisms is apparently due to the protein structure that determines the accessibility of the substrate to the active center in the enzyme, formed by the triad Cys^72^, His^110^, and Asp^125^. 

The incorporation of AHBA starter analogous units by *S. hygroscopicus* produced compounds similar to geldanamycin with potential antitumor activity [33]. However, none of them improved the antitumor activity of the native geldanamycin. 

### 2.4. Post-Polyketide Modifications

The pro-geldanamycin intermediate is finally modified by a series of polyketide modifications that include hydroxylation at C-17 and oxidations at C-18 and C-21, followed by methylation of the hydroxyl group at C-17, introduction of the carbamoyl group at C-7 and finally formation of the double bond between C-4 and C-5. The C-17 hydroxylation and the C-18 and C-21 oxidations have remained unclear for many years; these reactions might be performed by at least one flavin-dependent oxidase, encoded by *gdmM* [15]. The O-methyltransferase that introduces a methyl group at the hydroxyl formed at C-17 was not initially found in the geldanamycin gene cluster but later Yin et al. [15] found a putative methyltransferase gene, *gdmMT*, located 17 kb away from the main geldanamycin gene cluster in *S. autolyticus*. The involvement of the *gdmMT* gene in the methylation of the hydroxyl group at C-17 was supported by in vivo and in vitro experiments. The O-carbamoyl transferase encoding gene, *gdmN*, cloned from *S. hygroscopicus* 17997 is very similar to the orthologous genes of other geldanamycin producers [34]. The *gdmN*-disrupted mutant synthesized C-7 decarbamoyl-geldanamycin confirming that the enzyme encoded by this gene is involved in the carbamoylation at C-7. Furthermore, these authors complemented the disrupted mutant with the wild type *gdmN* gene resulting in the formation of geldanamycin. Interestingly, the *gdmN* disrupted mutant was able to synthesize several novel geldanamycin compounds, including 4,5-dihydro-7-O-decarbamoyl-7-hydroxy-19-O-glycylgeldanamycin and 4,5-dihydro-7-O-decarbamoyl-7 hydroxygeldanamycin. These novel compounds showed less activity against human tumor cell lines but instead they showed better solubility [34]. Finally, desaturation resulting in the formation of the C-4/C-5 double bond was catalyszd by a P450 monooxygenase encoded by *gdmP* (see below).

## 3. Geldanamycin Gene Clusters in Different *Streptomyces* Species 

Similar geldanamycin gene clusters have been found in other *Streptomyces* species (Table 1). Recent studies using different geldanamycin producers, as S. *hygroscopicus* sub. *duamyceticus* JCM4427 [20], have shed light on the post-polyketide modification reactions, although the use of different names for the same gene makes it difficult to compare the findings of different authors.

Two of the genes, named *gel*1 and *gel*7 by these authors (equivalent to *gdmL* and *gdmM*, respectively), encode flavin dependent oxygenases. Disruption of *gel*1 did not affect geldanamycin production, indicating that *gel*1 has no role in geldanamycin biosynthesis, whereas disruption of *gel*7 led to the formation of 17-demethoxy-reblastatin, an ansamycin containing a benzoquinone nucleus, suggesting that *gel*7 is involved in an oxidation of the benzoquinone ring. Complementation of the *gel*7 disrupted mutant with the wild type *gel*7 gene re-established the geldanamycin production. Shin et al. [20] proved that the hydroxylations at C-17 and C-21 are previous to the C-7 O-carbamoylation. They also described that *gel*16 (equivalent to *gdmP*), that is linked to a ferredoxin gene, encodes a P450 oxygenase, containing an heme-pocket. Disruption of the *gel*16 gene results in the formation of 4,5 dihydrogeldanamycin, and geldanamycin production was restored by complementation with a wild type *gel*16 gene suggesting that *gel*16 is involved in the formation of the geldanamycin C-4/5 double-bond. This was formally demonstrated by Rimal et al. [37] obtaining recombinant Gel-16 protein in *E. coli* and using it to perform the in vitro conversion of 4,5-dihydrogeldanamycin to geldanamycin. In silico protein–protein docking studies were performed by these authors to identify putative ferredoxin and ferredoxin reductases electron transporters cooperating with Gel16, involved in the dehydrogenation step. Although no hydroxylated intermediates were found in the *gel*16- disrupted mutant, the authors do not exclude that a hydroxylated intermediate is involved in the process of the double-bond formation.

In another geldanamycin producer, *S. autolyticus* JX-47, the complete genome consists of a 10 Mb lineal chromosome and 7 circular plasmids [22]. This strain was obtained from a laboratory at Yunnan (China) as an autolytimycin producing strain. Later, it was shown that this strain also produced geldanamycin, and a geldanamycin gene cluster was isolated in a bacterial artificial chromosome (BAC) library [22]. From this library, a 250 kb contiguous region of *S. autolyticus* JX-47 DNA, that included the complete geldanamycin cluster, was subcloned. In this strain, the central polyketide region of the geldanamycin gene cluster is 99% identical to that of the *S. hygroscopicus* 17997 strain and the encoded proteins range from 81% to 100% identity in amino acid sequence to the orthologous proteins of *S. hygroscopicus*. All genes of the geldanamycin cluster in *S. autolyticus*, have similar organization to those of *S. hygroscopicus* NRRL3602, except *gdmL* and *gdmX*, which are absent from the core region (Figure 2, Table 2). 

## 4. Regulatory Genes in the Geldanamycin Gene Cluster

**Figure 3:**
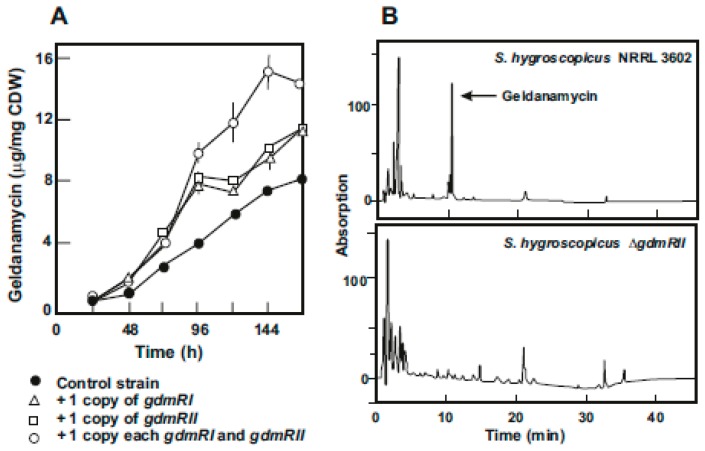
Effect of the regulatory genes on geldanamycin production. (**A**) Production of geldanamycin by *S. hygroscopicus* var. *geldanus* NRRL3602 transformed with the empty plasmid pRA (black circles), and the pRA plasmid [38] carrying insertions with the genes *gdmRI* (white triangles), *gdmRII* (white squares), and *gdmRI+gdmRII* (white circles). (**B**) HPLC analysis of geldanamycin production by *S. hygroscopicus* var. *geldanus* NRRL3602 (upper panel) and *S. hygroscopicus* Δ*gdmRII* (lower panel) in which *gdmRII* was deleted. The arrow indicates the geldanamycin peak.

In the early studies on geldanamycin biosynthesis, Rascher et al. [18] found that in the geldanamycin gene cluster of *S. hygroscopicus var. geldanus* there were several putative regulatory genes. A similar finding was reported later in the cluster of *S. hygroscopicus* 17997 [35]. These authors cloned and characterized two geldanamycin regulatory genes, named *gdmRI* and *gdmRII*; both genes encode LAL type regulators which contain a Walker motif (ATP/GTP binding site) in the amino terminal region and a helix-turn-helix binding domain in the carboxyl end. Both GdmRI and GdmRII act as positive regulators of the geldanamycin biosynthesis as shown by gene disruption and complementation studies. In an independent parallel work, we isolated a mutant of *S. hygroscopicus* var. *geldanus* NRRL3602 deleted in *gdmRII* and observed that this mutant was completely blocked in geldanamycin biosynthesis (Figure 3). 

Both, the results of He et al. [35] and our own experiments, indicate that disruption of one of the two LAL regulatory genes completely abolished geldanamycin biosynthesis. This means that both, *gdmRI* and *gdmRII* genes, are independent and are not simply duplicated regulatory genes. He et al. [35] showed that expression of these two genes is independent of each other; i.e., inactivation of one of these genes did not affect expression of the other one. These authors proved that inactivation of the *gdmRI* or *gdmRII* gene suppresses expression of the polyketide biosynthetic genes but does not affect transcription of post-polyketide modification genes and we confirmed that deletion of *gdmRII* resulted in lack of production of geldanamycin (Figure 3B). Using *S. hygroscopicus* NRRL3602, we amplified in an integrative monocopy vector either *gdmRI*, *gdmRII*, or both. The results indicated that production of geldanamycin increased about 33% with an additional copy of either *gdmRI* or *gdmRII*, but an important increase (more than 100%) was found when the wild-type strain was transformed with a combination of *gdmRI* and *gdmRII* (Figure 3A).

Later Kim et al. [36] studied the regulatory genes located close to the geldanamycin gene cluster from a different strain, *S. hygroscopicus* var. *duamyceticus* JCM4427, obtained from the Japanese culture collection of microorganisms (Table 1). These authors found up to five putative regulatory genes near the geldanamycin cluster; three of which were located downstream of the *gdm* cluster; these three genes, named *gel*14, *gel*17, and *gel*19, were characterized in detail [36]. Genes *gel*14 and *gel*17 encode LAL-type regulators that are identical to GdmRI and GdmRII described in *S. hygroscopicus* 17997 [35]. The identity between the two LAL-type regulator Gel14 and Gel17 is low (26%) and this explains why these two regulators are not functionally equivalent and work independently of each other in controlling geldanamycin biosynthesis [35]. On the other hand, *gel*19 encodes a TetR-type transcriptional factor that acts as a positive regulator of geldanamycin biosynthesis. The *gel*19 regulator belongs to the well-known family of TetR transcriptional factors, which are characterized as homodimers with an DNA binding helix-turn-helix domain at their N-terminal region and a ligand binding domain in the C-terminal end [39,40]. Studies of these regulatory genes and comparison of gene expression in mutants disrupted in each of these genes provide evidence indicating that regulation in strain *S. hygroscopicus* JCM4427 is somehow different from that found previously in *S. hygroscopicus* 17997. Indeed, in strain *S. hygroscopicus* JCM4427, *gel*17 and *gel*19 are required for expression of the *gel*14 LAL-type regulator and of the common promoter of the *gelA* genes (equivalent to *gdmA*, encoding the three polyketide synthases). Mutants in *gel*14 were able to express normally *gel*17 and *gel*19, whereas mutants in *gel*17 and *gel*19 did not express the LAL regulator *gel*14 or the polyketide synthase gene. In conclusion, the available evidence suggests that there is a cascade in which expression of *gel*14 is controlled by *gel*17 and *gel*19 and, in turn, *gel*14 is required for expression of GelA and controls initiation of transcription of *gel*8 (carbamoyl transferase) and *gel*16 (P450 oxygenase). 

LAL-type regulators similar to Gel14 and Gel17 are encoded in many other polyketide gene clusters as, for instance PimM of the pimaricin producer *Streptomyces natalensis* [41,42], FkbN of the tacrolimus producers *S. tsukubaensis* and *S. hygroscopicus* var. *ascomyceticus* [43,44] or FscRI in the candicidin producer *Streptomyces griseus* [32,45], among others. 

### Regulatory Genes in the Geldanamycin Gene Cluster of Streptomyces Autolyticus: Regulation of the Distant Unrelated Elaiophylin Cluster

The complete genome of *S. autolyticus* CGMCC 0516, isolated from a soil sample of Yunnan province (China), was reported recently [23]. It encoded 57 putative secondary metabolite clusters, many of them similar to known gene clusters in other *Streptomyces* species. These include the geldanamycin, autolytimycin, and reblastatin biosynthetic clusters, located in the left arm of the chromosome, and the cluster for the macrodiolide elaiophylin in the right end of the chromosome. The three first compounds are benzoquinone-type ansamycins and all of them are able to bind the Hsp-90 chaperone. Previous studies on the target of the proteins encoded by the three genes, *gel*14, *gel*17, and *gel*19 of the geldanamycin gene cluster in *S. hygroscopicus* [36] showed that these genes were cluster situated regulatory genes, as occurs in other actinomycetes [46], that affected specifically geldanamycin biosynthesis. However, recent evidence in *S. autolyticus* on the role of GdmRIII (*gel*19), encoding a TetR regulator (see above) revealed that this regulator controls not only geldanamycin biosynthesis but also formation of elaiophylin, which is an unrelated antibiotic encoded by a gene cluster located in the distal end of the genome with respect to the geldanamycin gene cluster [47].

The regulatory role of GdmRIII on the biosynthesis of autolytimycin and on elaiophylin biosynthesis was investigated using a knock-out mutant in the *gdm*RIII gene. This mutant produced only 19% of the geldanamycin-type compounds in relation to the wild type but, showed a 3.2-fold increase in the formation of three new compounds that were initially thought to be geldanamycin-type derivatives. Surprisingly, when the molecular mass and the nuclear magnetic resonance spectra were determined, they were found to be elaiophylin and elaiophylin derivatives, namely 11-methyl-elaiophylin and 11,11 dimethyl-elaiophylin. Complementation of the *gdmRIII* mutant with the wild-type allele restored production of geldanamycin up to 80% in relation to the parental strain and reduced the levels of the three elaiophylin derivatives to the normal levels as in the parental strain. These results clearly indicate that although the *gdmRIII* gene is located in the geldanamycin cluster, it regulates a far distant unrelated cluster; this agrees with recent findings of regulation of distant clusters by a single transcription factor [47,48,49]. The *gdm*RIII encoded protein was expressed in *E. coli* and was found to bind to the upstream regions of several genes in the geldanamycin gene cluster, namely *gdm*M, *gdm*N, and *ela*F in the elaiophylin gene cluster [47]. 

## 5. Engineering of Polyketide Biosynthesis in Geldanamycin Producing Strains

In the last decades the availability of genome sequences of different *Streptomyces* species has provided evidence indicating that many *Streptomyces* contain type I polyketides gene clusters [50,51]. The biosynthesis of these polyketides requires acyl-CoA precursor units including malonyl-CoA methylmalonyl-CoA, methoxymalonyl-CoA, allylmalonyl-CoA, among others. When these polyketides are produced simultaneously, they compete for the available common precursors. In the *S. hygroscopicus* 17997 strain, Li et al. [52] disrupted two polyketide genes different from that of geldanamycin and observed distinct effects; disruption of the polyketide *cos-10* gene did not affect geldanamycin production but in contrast, disruption of the polyketide *pg-10* gene doubled the production of geldanamycin supporting the conclusion that there is competition between the biosynthesis of geldanamycin and that of Pg-10 polyketide, which does not occur with other polyketides. At this time, the molecular mechanism of the specific competition between geldanamycin and polyketide Pg-10 is not yet clear.

Several important efforts have been made to obtain novel geldanamycin derivatives by modification of the biosynthetic pathway [53]. Moreover, Kim et al. [13] obtained, by combined mutagenesis of the geldanamycin PKS and modification of the tailoring enzymes, a C15-hydroxylated, C17-demethoxy non-quinone geldanamycin analogue, DHQ3, that has 4.6-fold higher Hsp90 ATPase activity inhibition than geldanamycin

## 6. Phosphate Control of Geldanamycin Biosynthesis 

Growth of the *Streptomyces* species and the biosynthesis of antibiotics and other secondary metabolites is controlled by the concentration of phosphate in the medium [54]. However, there are important differences in the sensitivity to phosphate of the biosynthesis of distinct secondary metabolites [55,56,57].

**Figure 4:**
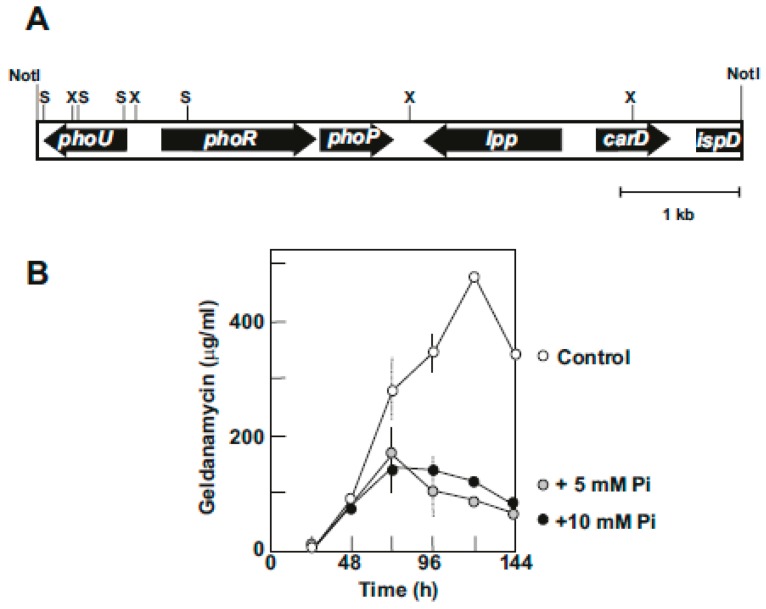
Phosphate control of geldanamycin production. (**A**) Organization of the *pho* genes and the surrounding region in a NotI DNA fragment of *S. hygroscopicus* var. *geldanus* NRRL 3602. Restriction sites for XhoI (X) and SalI (S) are indicated. (**B**) Production of geldanamycin in SPG medium (white circles) and SPG medium supplemented with 5 mM (gray circles) and 10 mM inorganic phosphate (black circles).

Our studies on the effect of inorganic phosphate in production of geldanamycin by *S. hygroscopicus* var. *geldanus* NRRL3602 showed that the biosynthesis of geldanamycin is highly sensitive to inorganic phosphate (Figure 4B). In soy-peptone-glucose (SPG) medium, which supports high geldanamycin production (above 500 μg/mL at 120 h), inorganic phosphate at 5 mM or higher concentration reduces geldanamycin production by 80%.

### 6.1. Cloning of the phoU- phoRP Gene Cluster of S. hygroscopicus NRRL3602

Phosphate control of both primary metabolism and the biosynthesis of secondary metabolites is mediated by the two components PhoR-PhoP system [58,59].

A cosmid library of total DNA of *S. hygroscopicus* var *geldanus* in the superCos1 vector (Stratagene) was constructed and screened by hybridization with a 1.2 kb DNA probe containing the *phoRP* genes of *S. coelicolor* [60]. A cosmid, Cos17d1, showing a high hybridization signal was selected and a 7.0 kb fragment of the insert was subcloned in pBluescript and sequenced. Five orfs were found in this insert that include *phoR*, *phoP*, *phoU*, *lpp*, *carD*, and the incomplete *ispD* gene (Figure 4A).

*phoR-phoP* were located in the opposite orientation to *phoU*; the three genes are expressed from a divergent promoter region as occurs in other *Streptomyces* species [56,57]. The *lpp* gene encode a lipoprotein, the *carD* gene encodes a CarD family transcriptional regulator that binds RNA polymerase, and the incomplete *ispD* encodes a cytidyltransferase. 

The *S. hygroscopicus* sensor kinase PhoR is a protein of 432 amino acids that contains the characteristic Boxes H, N, D/F, and G [61] present in all PhoR proteins. PhoR has a high identity in amino acid sequence to the orthologous proteins of *S. coelicolor* (85%), *S. avermitilis* (86%), and *S. natalensis* (88%). 

The response regulator PhoP has 223 amino acids and is 98% identical to the homologous protein of *S. coelicolor* and *S. avermitilis*. PhoP is extremely well conserved in all *Streptomyces* species [62] supporting its important role on the PhoR-PhoP mediated response [63]. The PhoP protein has the conserved aminoacids Asp^6^ and Asp^49^ in the amino terminal region, lysine K^98^ for phosphorylation, as part of the receiver domain, and a DNA binding domain (DBD) in the carboxyl terminal end (amino acids 190-201). The distance between *phoR* and *phoP* is only 5 nucleotides, strongly suggesting that these two genes are co-transcribed as reported previously in *S. coelicolor* [60,64].

The divergent *phoU* gene encodes a 275 amino acids protein 92% identical to the PhoU of *S. natalensis* and 93% to those of *S. coelicolor* and *S. avermitilis*. In *S. coelicolor*, *phoU* encodes a modulator of the PhoRP expression that exerts self-control of phosphate regulation [64]. In addition, the *pstS* gene, encoding the high affinity phosphate transport was cloned by hybridization with a probe of the orthologous *S. coelicolor* gene. 

### 6.2. Disruption and Characterization of the PhoP Gene: Effect on Growth and Geldanamycin Production

In order to study the effect of PhoP on growth and production of geldanamycin, the PhoP gene was disrupted by an apramycin resistance gene in cosmid Cos17-D1 in *E. coli* and the disrupted *phoP* gene was then replaced in *S. hygroscopicus* by the REDIRECT technique. The mutation was confirmed by PCR amplification and sequencing of a 2.6 kb *XhoI* fragment containing the gene replacement. The nucleotide sequence of this fragment confirmed the disruption of *phoP*.

To study the effect of the inactivation of *phoP* on phosphate utilization, growth and geldanamycin production, the *phoP* mutant was grown in SPG, a medium that is an excellent nutrient for polyketide antibiotics production. 

Interestingly, the disruption of *phoP* had a very strong effect on growth of *S. hygroscopicus*. The mutant was unable to grow in SPG medium in the absence of phosphate supplementation, although the SPG medium contains organic phosphate i.e,. it behaved as an inorganic phosphate auxotroph. When SPG medium was supplemented with 2.5, 5, 9, or 15 mM phosphate, there was an increasing recovery of growth at the 5-, 9- and 15-mM concentration, but the mutant showed very limited growth in 2.5 mM phosphate supplemented medium. This strong dependence of growth has not been observed in *S. coelicolor* or *S. natalensis phoP* mutants using the same medium. These two *Streptomyces* species grow in the absence of inorganic phosphate because they were able to hydrolyze organic phosphate present in the soy-peptone medium, whereas *S. hygroscopicus* seems unable to do so. This might be due to the absence of some extracellular phosphatases in *S. hygroscopicus*, as described also in *S. tsukubaensis* [62]. Although *S. hygroscopicus phoP* mutant grows well at 5 mM phosphate, the production of geldanamycin was higher at 15 mM which is a phosphate concentration normally inhibitory for antibiotic biosynthesis, as is the case in *S. natalensis* for pimaricin biosynthesis [55], suggesting that the *phoP* mutant is deregulated in PhoP mediated control of geldanamycin biosynthesis.

### 6.3. Identification of PhoP Binding Sequences in S. hygroscopicus Genes

**Figure 5:**
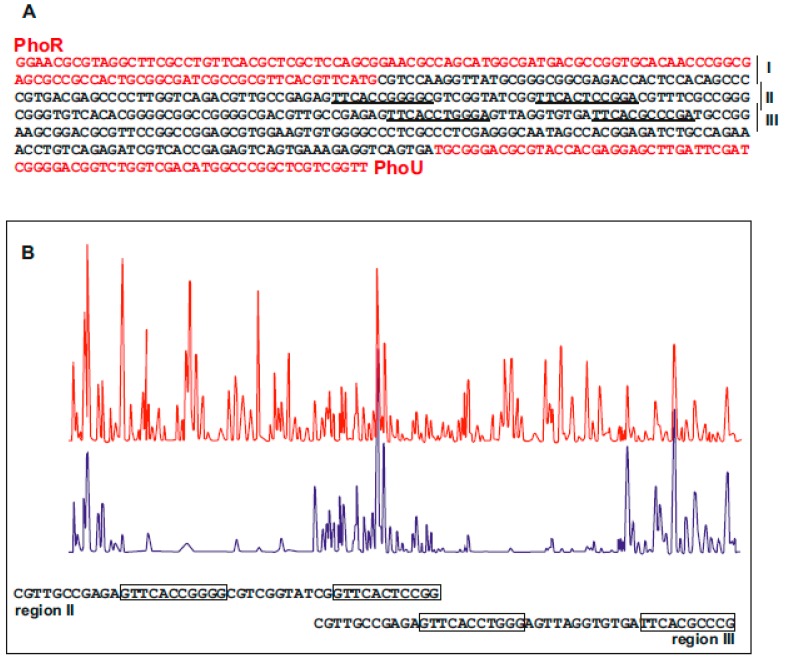
Localization of PHO boxes in the intergenic region *phoRP-PhoU* of *S. hygroscopicus* var. *geldanus* NRRL 3602. (**A**) Sequence of the intergenic region *phoRP-phoU* showing in red the nucleotides belonging to the *phoP* and *phoU* genes. The regions I, II, and III used in EMSA analysis are indicated. (**B**) Footprinting analysis of DNA containing regions II and III. The upper line (in red) corresponds to the parental strain DNA without the PhoP^DBD^-GST protein. The lower line (in blue) corresponds to the same DNA sequence protected by addition of PhoP^DBD^-GST protein. PhoP^DBD^-GST protein (2 μM) was used in the assay as described by Sola-Landa et al. [66].

It is known that PhoP binds an 11 nucleotides direct repeat sequence in the genome of different *Streptomyces* species [56,60,65]. Although the genome of *S. hygroscopicus* var *geldanus* was not available, we searched for PhoP binding boxes in the promoter regions of *phoU*, *phoRP*, and *pstS* genes that were cloned as indicated above. Bioinformatic analysis of the promoter regions of these genes allowed us to identify the putative PHO boxes in these promoters. The intergenic sequence *phoRP-phoU* (Figure 5A) was divided in three segments (regions I, II, III) that were subjected to EMSA analysis using *S. coelicolor* PhoP^DBD^ linked to-GST (0.2 to 3.2 pMols) obtained as a recombinant protein in *E. coli* [60]. The results showed a clear retardation in the mobility assays of the DNA fragments in regions II and III but no gel shift was observed in region I. Bioinformatic analysis of these regions and of the *pstS* gene promoter showed the presence of putative PHO boxes for PhoP binding. To confirm the nature of these PhoP binding sequences, a DNAse-footprinting analysis was performed on a 389 bp DNA fragment carrying the regions II and III upstream of the *phoRP-phoU* promoter and PhoP^DBD^-GST protein at 2 μM concentration. A DNA protection footprinting analysis showed two protected regions (Figure 5B) containing the 11-nucleotides repeats (PHO boxes) detected also by bioinformatic analysis. Alignment of the PHO boxes of *phoU-phoRP* and *pstS* genes provided a DNA-binding sequence (GTTCACCCGCC), similar to that of *S. coelicolor* and *S. avermitilis* [56], although with minor differences in the frequency of alternative nucleotides. 

In summary, our studies on the phosphate control of geldanamycin biosynthesis indicate regulation by the phosphate concentration in the medium as occurs with many other polyketides, but it is clearly more sensitive to the inorganic phosphate concentration than other polyketides.

## 7. Future Outlook

The ansamycin geldanamycin and its derivatives have great potential as antitumor agents due to their interaction with the Hsp-90 chaperone in human cells. Clinical assays have shown that geldanamycin produces hepatoxicity and side effects affecting healthy human celIs. Novel geldanamycin derivatives obtained by metabolic engineering have been developed. Research on novel strains producing less toxic geldanamycin analogs is required. Moreover, at this time geldanamycin is an important tool for research in human tumorigenesis, tumor cell dissemination and cell apoptosis [67]. Biosynthesis of geldanamycin is well known but the regulatory mechanisms that control expression of geldanamycin biosynthesis genes needs further research. There are several regulatory mechanisms that control gene expression limiting the production of this antitumor agent; expression of the three positive regulators *gdmRI*, *gdmRII*, and *gdmRIII* need to be optimized to obtain maximal geldanamycin production; the nucleotide sequences recognized by each of these transcriptional regulators have to be elucidated in order to search for additional target genes. Geldanamycin biosynthesis is extremely sensitive to the phosphate concentration in the culture medium. Phosphate is limiting for growth but, at high concentration, strongly represses the biosynthetic genes and therefore it has an important regulatory role both in growth and geldanamycin production. Moreover, other pleiotropic regulators that control nitrogen metabolism, carbon source utilization, and coordination of metabolism are not known in these *Streptomyces* species and need to be investigated. The balance between utilization of nitrogen and carbon sources is very important in the biosynthesis of geldanamycin because of the involvement of the mC_7_N precursor unit that requires an amino group to form the AHBA starter unit. The availability of the genome sequence of both *S. hygroscopicus* XM-201 [21] and *S. autolyticus* CGMCC 0516 [23] provides new useful “omics” information to dissect the genome of these geldanamycin producing strains. Transcriptomic analysis of expression of different genes involved both in the biosynthesis of structural components of geldanamycin and in the control of the biosynthesis of this antitumor agent needs to be emphasized. Finally, metabolomic studies leading to removal of side-product contaminants are also required to further increase the production of geldanamycin and related. 

## 8. Conclusions

The biosynthesis of geldanamycin in different producer strains is regulated by several transcriptional factors. Three of them *gdmRI*, *gdmRII* and *gdmRIII* are situated close to the geldanamycin gene cluster. Moreover, the biosynthesis of geldanamycin is strongly regulated by phosphate; this regulation is mediated by the two-component system PhoR-PhoP. The PhoP binding sequence in *S. hygroscopicus* have been identified in this article.

## Figures and Tables

**Table 1:** Strains producers of geldanamycin.

Streptomyces Strains	Reference
*Streptomyces hygroscopicus var geldanus NRRL3602*	[18]
*Streptomyces hygroscopicus 17997*	[19]
*Streptomyces hygroscopicus sub. duamyceticus JCM 4427*	[20]
*Streptomyces hygroscopicus XM 201*	[21]
*Streptomyces autolyticus JX-47*	[22]
*Streptomyces autolyticus* CGMCC 0516,	[23]
*Streptomyces cameroonensis sp*.	[24]
***Streptomyces* species containing geldanamycin biosynthesis genes identified bioinformatically**	
*Streptomyces violaceusniger* Tu 4113 *Streptomyces* sp. RTd22 *Streptomyces lydicus* strain 103 *Streptomyces rapamycinicus* NRRL 5491 *Streptomyces iranensis* *Streptomyces albus* DSM 41398 *Streptomyces bingchenggensis* BCW-1	Cited in [23]

**Table 2:** Genes in the geldanamycin cluster and function of the encoded enzymes.

Genes	Function	Reference
*gdmMT*	O-methyltransferase	[15]
*gdmL = gel1*	Flavin-dependent oxygenase	[20]
*gdmX*	Unknown	[18]
*gdmAI, AII, AIII = gelAI, AII, AIII*	Polyketide synthases I, II and III ^1^	[18] ^2^
*gdmF*	Amide synthase	[33]
*gdmM = gel7*	Flavin-dependent oxidase	[20]
*gdmN = gel8*	Carbamoyl transferase	[20,34]
*gdmH*	Methoxymalonyl-ACP biosynthesis	[18]
*gdmI*	Methoxymalonyl-ACP biosynthesis	[18]
*gdmJ*	Methoxymalonyl-ACP biosynthesis	[18]
*gdmK*	Methoxymalonyl-ACP biosynthesis	[18]
*gdmG*	O-methyl transferase for methoxy-malonyl-ACP biosynthesis	[18]
*gdmRII = gel17*	LAL-type regulator	[35,36]
*gdmO* *^3^*	Amino dehydroquinate synthase	[18]
*gdmFx*	Ferredoxin	[20]
*gdmP = gel16*	P450 monooxygenase	[20]
*gdmRI = gel14*	LAL-type regulator	[35,36]
*gdmRIII = gel 19*	TetR-family positive regulator	[36]

^1.^ The GdmAI, AII, AIII proteins are translated from a putative monocistronic mRNA encoded by the *gdmA* gene. The original *gdm* gene designation was proposed by Rascher et al. [18]. ^2.^ Other gene designations (*gel* genes) correspond to articles on the characterization of the genes. ^3.^ Other genes for AHBA biosynthesis have been cloned in *S. hygroscopicus* 17997 by He et al. [19], but except the amino dehydroquinate synthase (*gdmO*) were not located in the geldanamycin cluster described by Rasher et al. [18].
